# Data of thermal degradation and dynamic mechanical properties of starch–glycerol based films with citric acid as crosslinking agent

**DOI:** 10.1016/j.dib.2016.04.012

**Published:** 2016-04-11

**Authors:** Paula González Seligra, Carolina Medina Jaramillo, Lucía Famá, Silvia Goyanes

**Affiliations:** a LPMC. Dep. de Física – IFIBA (CONICET), FCEyN, UBA, Ciudad Universitaria, 1428 CABA, Argentina; b Instituto de Tecnología en Polímeros y Nanotecnología ITPN (UBA-CONICET), FCEyN, UBA, Av. Las Heras 2214, C1127AAQ, CABA, Argentina

**Keywords:** Starch, Citric acid, Biodegradable edible films, Thermal degradation, Dynamic mechanical properties

## Abstract

Interest in biodegradable edible films as packaging or coating has increased because their beneficial effects on foods. In particular, food products are highly dependents on thermal stability, integrity and transition process temperatures of the packaging. The present work describes a complete data of the thermal degradation and dynamic mechanical properties of starch–glycerol based films with citric acid (CA) as crosslinking agent described in the article titled: “Biodegradable and non-retrogradable eco-films based on starch–glycerol with citric acid as crosslinking agent” González Seligra et al. (2016) [1]. Data describes thermogravimetric and dynamical mechanical experiences and provides the figures of weight loss and loss tangent of the films as a function of the temperature.

**Specifications Table**

**Table:** 

Subject area	*Biomaterials*
More specific subject area	*Starch*–*glycerol based film with citric acid as crosslinking agent.*
Type of data	*Text file, figure.*
How data was acquired	*TGA*/*DTA equipment (DTG-60 Shimadzu themogravimetric analyzer) and Dynamic Mechanical Thermal Analyzer instrument (DMTA IV, Rheometric Scientific).*
Data format	*Analyzed, plotted.*
Experimental factors	*After the stabilization of the films on desiccators for 15 days, at 25* *°C, over saturated solution of NaBr (RH ~56%).*
Experimental features	*TGA was carried out from 40* *°C to 400* *°C at a rate of 10* *°C*/*min in a dry nitrogen atmosphere (flux rate: 30 mL*/*min).*
*Dynamic mechanical test were perform in the rectangular tension mode at 1* *Hz, 0.04% of strain, heating rate of 2* *°C*/*min and between −80 °C and 70* *°C.*
Data source location	*LP&MC, Dep. de Física-IFIBA (CONICET). Facultad de Ciencias Exactas y Naturales. Universidad de Buenos Aires. Ciudad Universitaria (1428), CABA, Argentina.*
Data accessibility	*Data are available in this article*

**Value of the data**

• Data presents detailed description of thermal degradation and dynamic mechanical behavior of starch–glycerol based films with citric acid as crosslinking agent.
• This data will be helpful for the scientific community that evaluates thermal stability and transition process of biodegradable materials for food packaging.
• Data allows the researcher to elucidate the action of citric acid in a polymer matrix.

## 1 Data

**Fig. 1:**
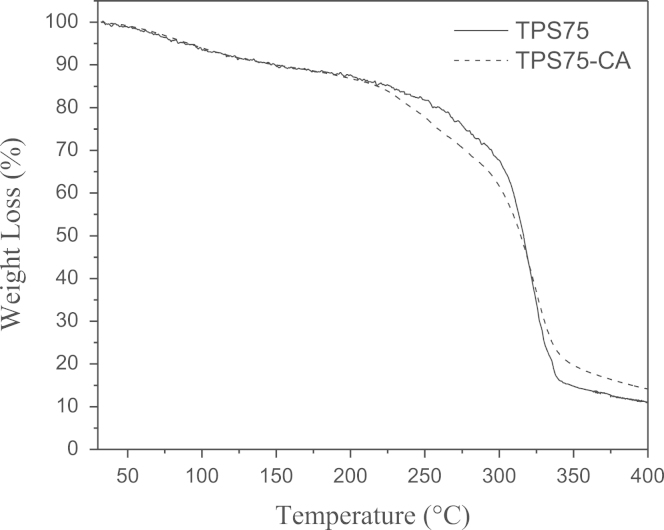
Thermogravimetric curves of TPS75 and TPS75-CA films.

**Fig. 2:**
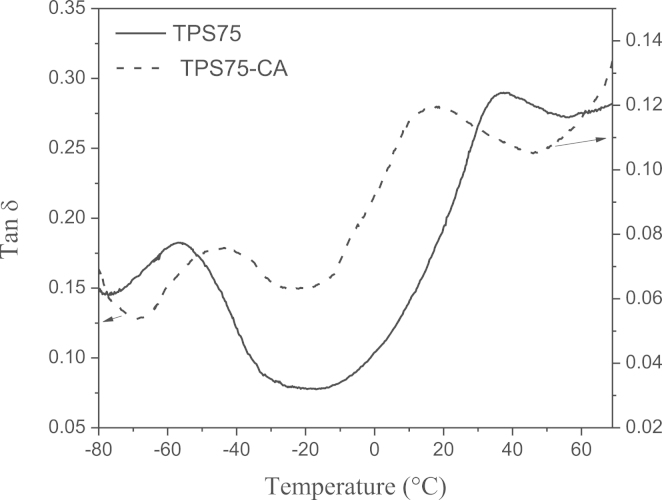
Loss tangent, tan*δ*, as a function of the temperature of TPS75 and TPS75-CA films.

Data presented in this work describes the thermal degradation and dynamic mechanical experiments on biodegradable and edible starch–glycerol based films with citric acid (CA) processed till 75 °C, described in the articles of González Seligra et al. [1]. These two characterizations are the aggregation of several properties previously investigated [1]. This data can improve the acknowledgments about the physicochemical properties of crosslinking starch systems. Weight loss as a function of the temperature of the films (Fig. 1), as well as, loss tangent (Fig. 2) is exposed.

## 2 Experimental design, materials and methods

The systems characterized in this data are biodegradable and edible films described in [1]. One system, namely TPS75 consists in starch, glycerol and distilled water; and, the other, is contains the same components and concentrations of TPS75 with the addition of 2 wt% of citric acid (CA). Both films was performed by casting and processed by till 75 °C.

### 2.1 Themogravimetric analyzer

The thermal stability of the films was obtained on a TGA/DTA equipment (DTG-60 Shimadzu *themogravimetric analyzer*). Approximately 5 mg of each sample was subjected to heating from 40 °C to 400 °C at a rate of 10 °C/min in a dry nitrogen atmosphere (flux rate: 30 mL/min). The weight loss of the samples was continuously recorded while the sample temperature was ramped at the constant heating rate. Then, the weight loss as function of the temperature was obtained. Three replicates were tested for each system and great repetitively was obtained.

Fig. 1 shows the thermogravimetric curves of starch–glycerol based film (TPS75) and starch–glycerol with CA system (TPS75-CA). Three main steps that take place in the range 40–150 °C, 150–280 °C and 280–350 °C are presented. The film with citric acid exhibits higher mass lost in the zones from 150 °C to 280 °C and between 280 °C and 350 °C. This system also shows great amount of mass at the end of the test (400 °C).

### 2.2 Dynamic mechanical properties

The experimental observations of possible relaxation processes of the evaluated films, as well as, the corresponding peaks temperatures, was carried out using a dynamic mechanical thermal analyzer (DMTA IV, Rheometric Scientific), in the rectangular tension mode at 1 Hz and heating rate of 2 °C/min. The temperature range of measurements was from −80 °C to 70 °C. The strain amplitude was 0.04% to assure that the mechanical response of the samples was within the linear viscoelastic range [2]. Samples dimensions were 20.0 mm×5.0 mm×0.20 mm (length, width and thickness, respectively). Three replicates were tested for each system with an important repetitively.

Fig. 2 shows the loss tangent, tan*δ*, as a function of the temperature for the films with and without citric acid. Two relaxation processes are exposed in both curves: one at ~−56 °C in TPS75 film and around −45 °C for TPS75-CA; and other at around 37 °C for TPS75 and ~17 °C for the film with citric acid. The films with CA showed a shift in both transition temperatures with respect to the films without CA. The first peak shifted to higher temperatures, while the latter toward lower temperatures.
